# Supplementary material for: Approximate Bayesian computational methods to estimate the strength of divergent selection in population genomics models
Source: J Comput Math Data Sci. Author manuscript; Available in PMC 2024 Apr 12. (PMC11014422; doi:10.1016/j.jcmds.2024.100091)
Supplement: 1 [file NIHMS1977881-supplement-1.pdf]

Appendix C

Supplemental figures.

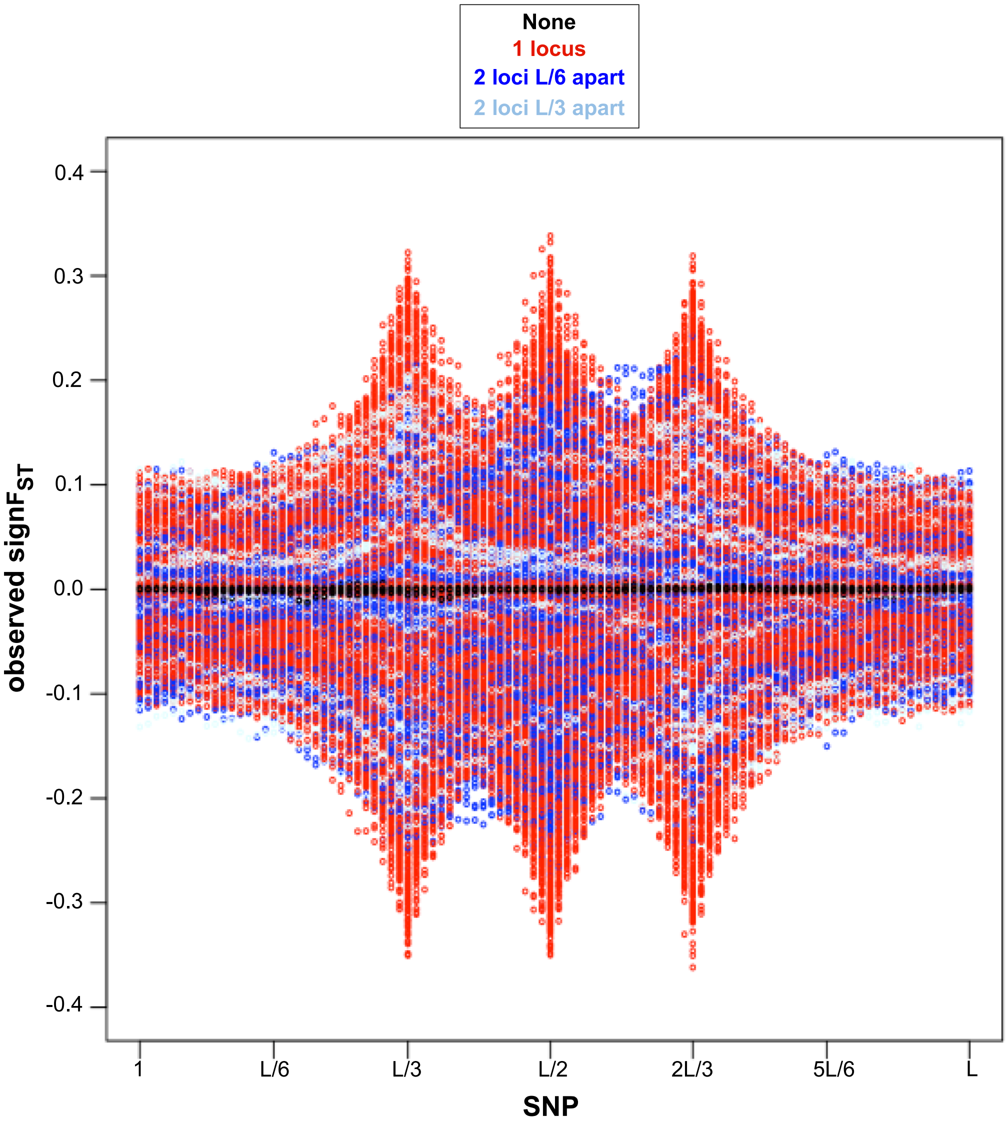

Fig. 11. SNP loci vs. $signF_{ST}$ summary statistics for the 1stmethod, i.e. without the initial outlier scan. Plots for each of 10,000 simulator output data sets assumed as observed under four of migration (m) and mode of reproduction (sex) combinations selected randomly with equal probability scenarios of loci under selection: none in black, one locus ($L/3$, or $L/2$, or $2L/3$) in red, two loci $L/6$ distance apart in navy blue ($L/3$ with $L/2$, or $L/2$ with $2L/3$), two loci $L/6$ distance apart in sky blue ($L/3$ with $2L/3$), as seen in **Fig. 2**. A visible pattern is shown of summary statistics values closest to 0 for no loci under selection, an increase in summary statistics values away from 0 at single locus under selection, and in-between the magnitude of summary statistics values away from 0 and increase in genetic hitchhiking effect for two loci under selection (lower observed frequency of recombination events between two loci under selection at a confined distance apart). For two loci under selection, an unclear difference in pattern is shown of summary statistic values for loci $L/6$ (navy blue) versus $L/3$ (sky blue) distance apart.

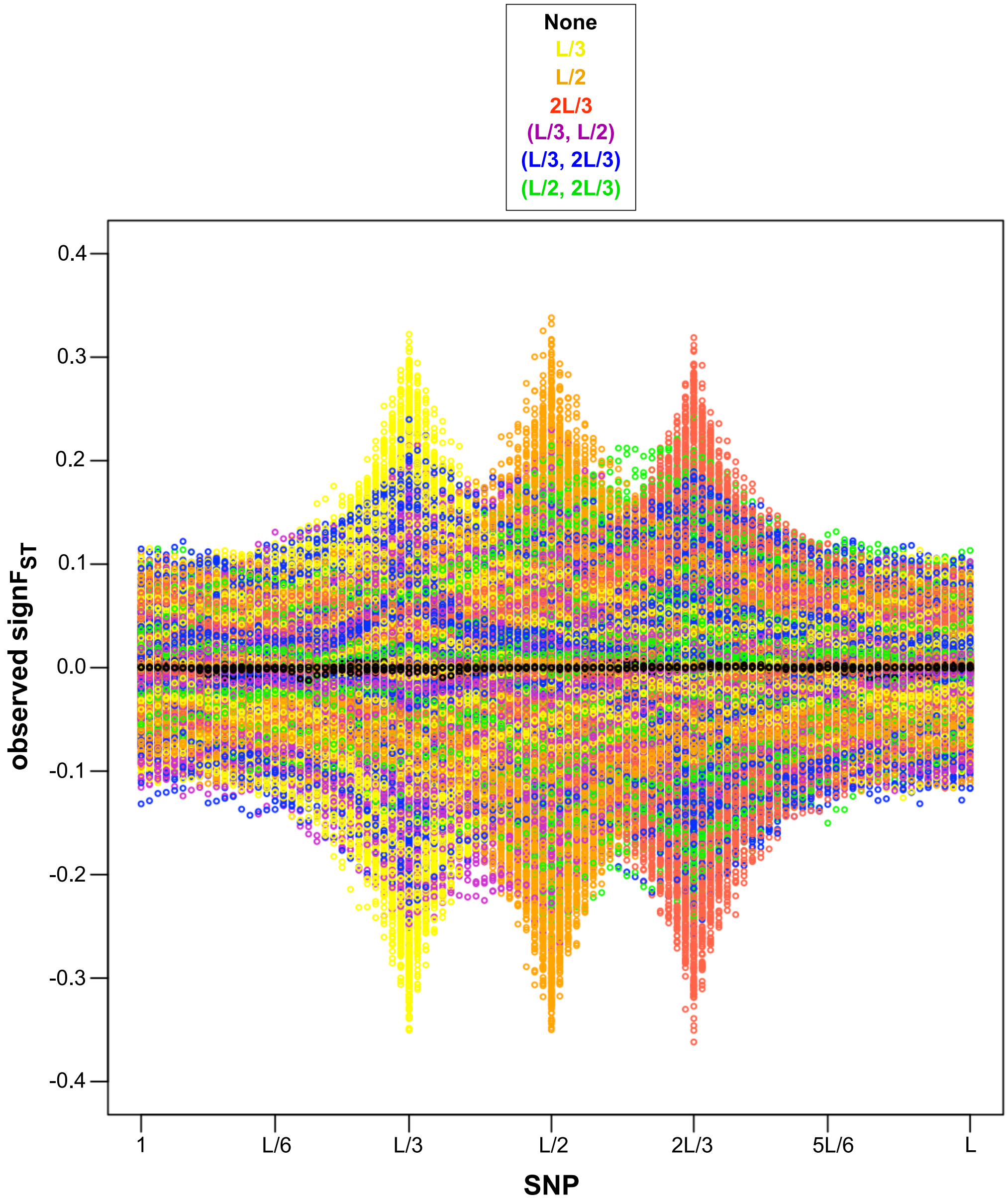

Fig. 12. SNP loci vs. $signF_{ST}$ summary statistics for the 1stmethod, i.e. without the initial outlier scan. Plots for each of 10,000 simulator output data sets assumed as observed under four of migration (m) and mode of reproduction (sex) combinations selected randomly with equal probability scenarios of loci under selection: none in black, $L/3$ in yellow, $L/2$ in orange, $2L/3$ in red, $L/3$ with $L/2$ in purple, $L/3$ with $2L/3$ in navy blue, and $L/2$ with $2l/3$ in green, as seen in **Fig. 2**. A visible pattern is shown of summary statistics values closest to 0 for no loci under selection, an increase in summary statistics values away from 0 at single locus under selection, and in-between the magnitude of summary statistics values away from 0 and increase in genetic hitchhiking effect for two loci under selection (lower observed frequency of recombination events between two loci under selection at a confined distance apart). For two loci under selection, an unclear difference in pattern is shown of summary statistic values for loci positions combinations of $L/3$ with $L/2$ (purple), versus $L/3$ with $2L/3$ (navy blue), versus $L/2$ with $2L/3$ (green).

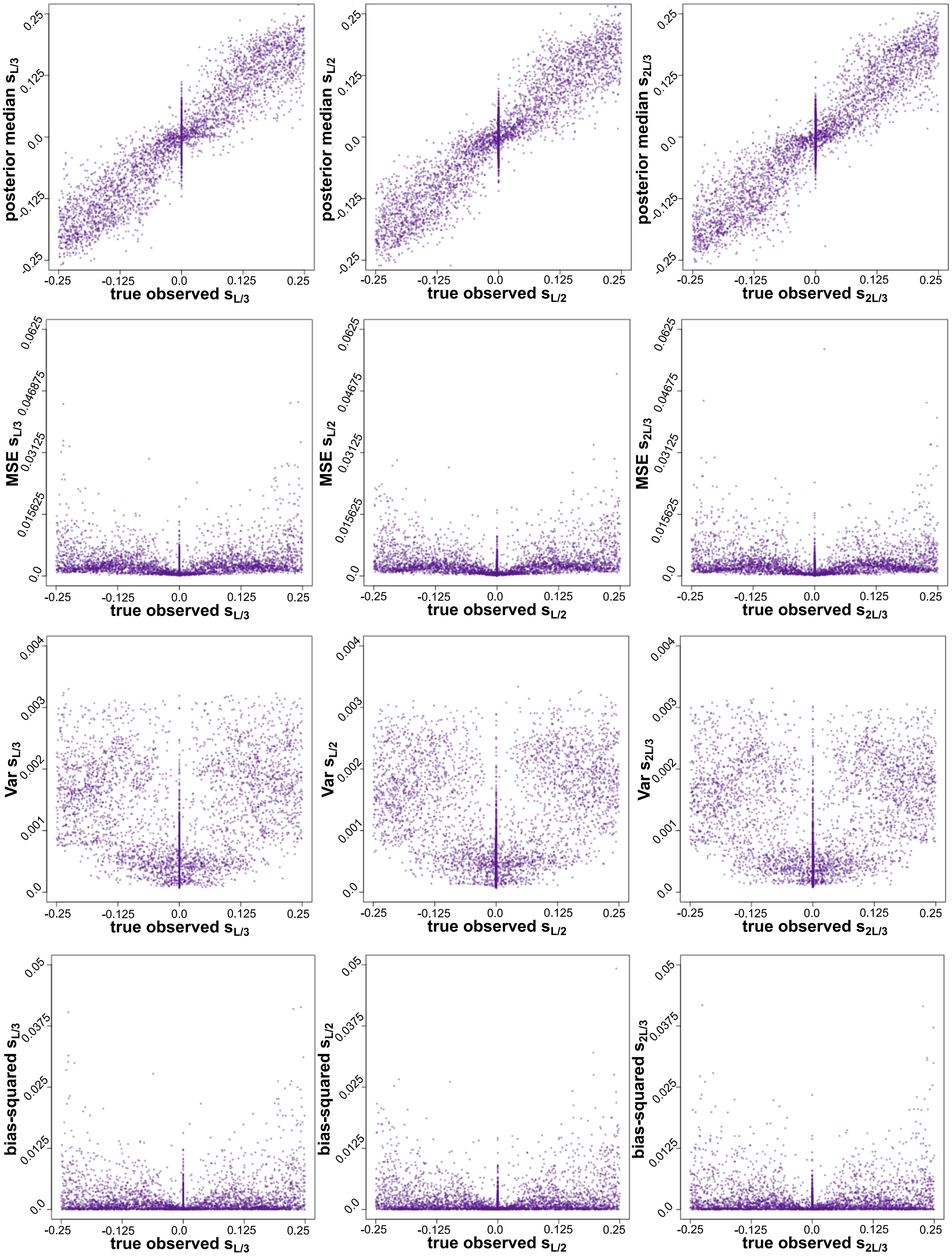

Fig. 13. True parameter value under which the observed parameter is generated (x-axis) vs. median, MSE, variance, bias-squared for $(L/3)^{\text{th}}$, $(L/2)^{\text{th}}$ and $(2L/3)^{\text{th}}$ SNP respectively for the 1^{st}method, i.e. without the initial outlier scan, from $n_{ABC} = 10,000$ ABC tests from $signF_{ST}$ summary statistics from **Algorithm 2** with Gaussian kernel.

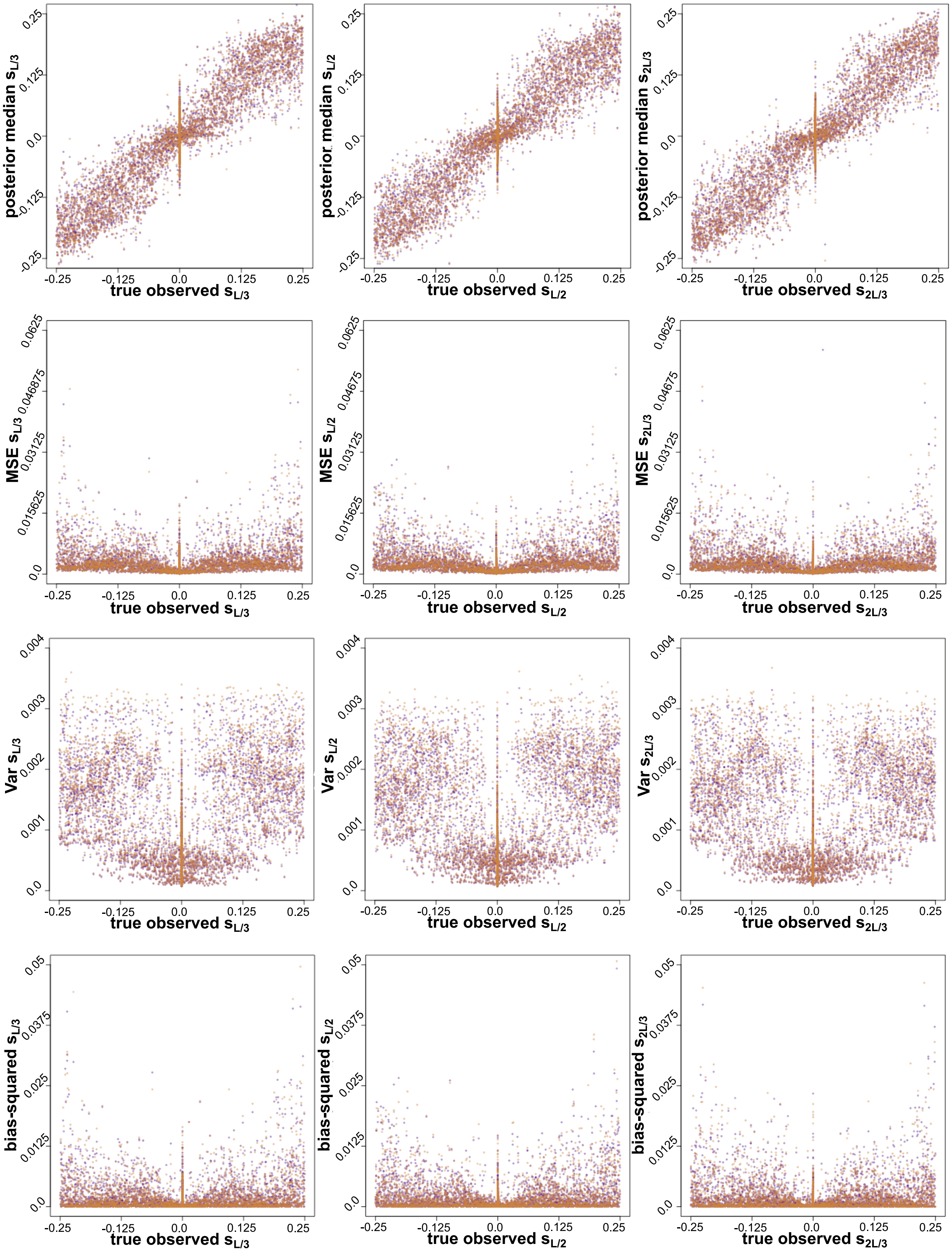

Fig. 14. True parameter value under which the observed parameter is generated (x-axis) vs. median, MSE, variance, bias-squared for $(L/3)^{\text{th}}$, $(L/2)^{\text{th}}$ and $(2L/3)^{\text{th}}$ SNP respectively for the 1[st]method, i.e. without the initial outlier scan, from $n_{ABC} = 10,000$ ABC tests from $signF_{ST}$ summary statistics from **Algorithm 2** with Gaussian kernel (purple) and Epanechnikov kernel (orange). Similar pattern observed with ABC-linear regression between the two kernels.

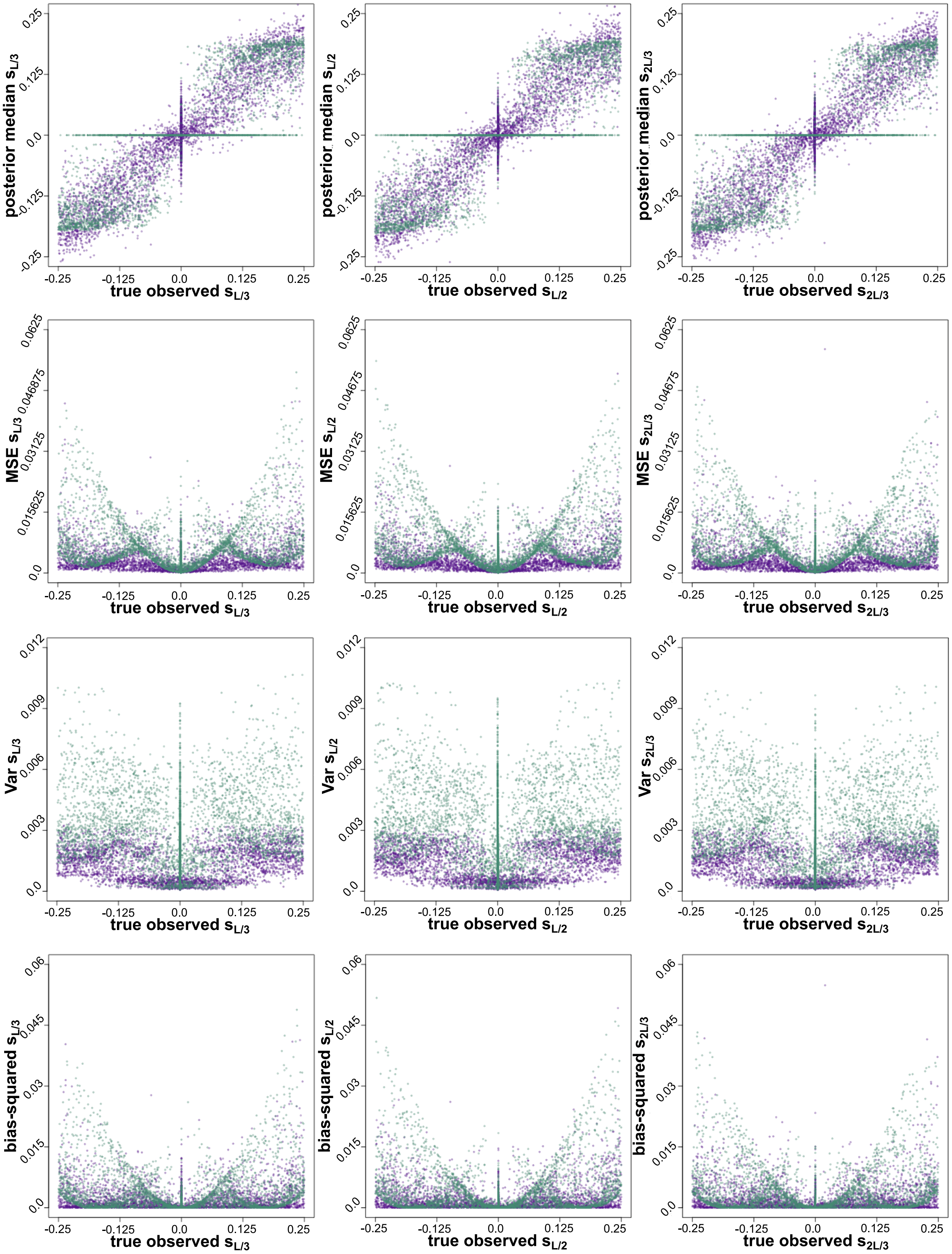

Fig. 15. True parameter value under which the observed parameter is generated (x-axis) vs. median, MSE, variance, bias-squared for $(L/3)^{\text{th}}$, $(L/2)^{\text{th}}$ and $(2L/3)^{\text{th}}$ SNP respectively for the 1^{st}method, i.e. without the initial outlier scan, from $n_{ABC} = 10,000$ ABC tests from $signF_{ST}$ summary statistics from **Algorithm 2** with Gaussian kernel (purple) and from **Algorithm 1** (green). ABC-linear regression with Gaussian kernel outperforms ABC-rejection.

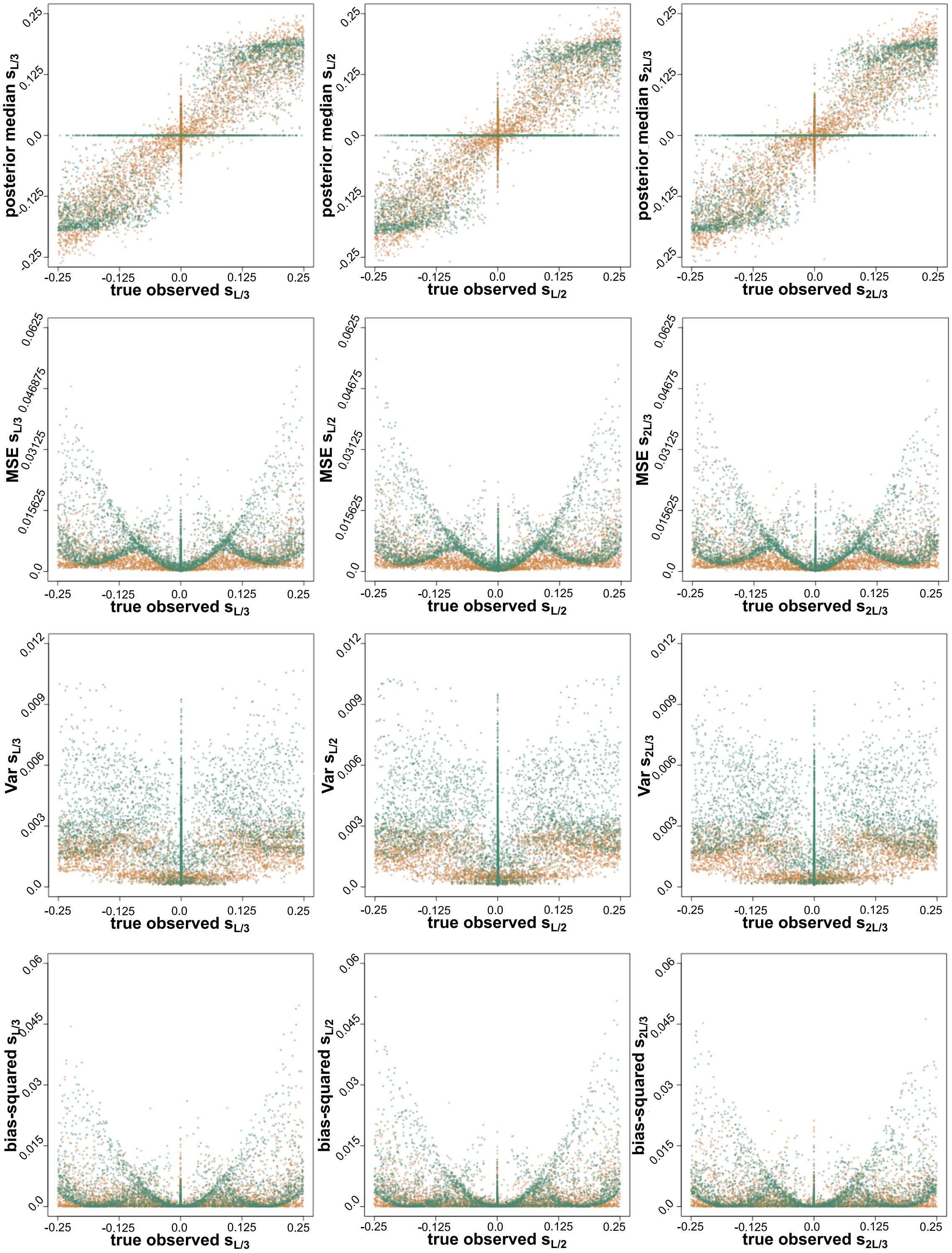

Fig. 16. True parameter value under which the observed parameter is generated (x-axis) vs. median, MSE, variance, bias-squared for $(L/3)^{th}$, $(L/2)^{th}$ and $(2L/3)^{th}$ SNP respectively for the 1^{st}method, i.e. without the initial outlier scan, from $n_{ABC} = 10,000$ ABC tests from $signF_{ST}$ summary statistics from **Algorithm 2** with Epanechnikov kernel (orange) and from **Algorithm 1** (green). ABC-linear regression with Epanechnikov kernel outperforms ABC-rejection.
